# Improving Mental Health of Medical Students through Movement, Art, and Interpersonal Relations

**DOI:** 10.1192/j.eurpsy.2024.1262

**Published:** 2024-08-27

**Authors:** E. Ajándék, V. Boros

**Affiliations:** ^1^Division of Integrative Medicine, Semmelweis University; ^2^Collection Management, Museum of Ethnography, Budapest, Hungary

## Abstract

**Introduction:**

Burnout, and symptoms of distress amongst medical students is becoming increasingly common due to the uncertainty of the Hungarian healthcare system. Change itself may be the cause of stress. Since the pandemic, the workload has been growing among health care workers. Anxiety is increasing even for beginner practitioners.

**Objectives:**

Our examination intends to improve the mental health of the students with a variety of methods to help them develop resilience towards everyday stress, such as:

1. increasing body awareness
2. exploring the inner drivers of vocations by self-esteem, worth symbols and emblems
3. supporting relationships and interpersonality

**Methods:**

We had advertised a monthly course in the mailing system of the students of the Semmelweis University (Neptun). Each occasion would go as far as 240 minutes in length. Selection criteria were: guaranteeing participation in the sessions. Any applicant suffering from mental health problems requiring medical attention, or the applicant regularly skipping occasions of the session would lead to his or her getting dismissed. Courses consists of musical aerob movement and receptive art therapy tools (exl. „Self-exhibition”). The aim was to interpret ones identity via images individually. In advance of the first occasion, the applicants were interviewed to talk about themselves their career, mental health and issues, why they want to participate in the sessions. The closing interviews are still in progress. The examination was permitted by the SE-RKEB.

**Method of examination:**

Qualitative: personal interviews, exploring talks about the artworks (“Self-Exhibition” collage), made during the course.

**Results:**

20 individuals started the course and 10 of them finished. The Body and Mind movements (Body Art - fusion of functional and breathing exercises, yoga and therapeutic exercises) has proven to have great importance throughout the session. The prescribed length of it in time was the third of each occasion. The joint analysation and interpretation of various artworks, images, visual narratives, even, the discussion of experiences in form of structured group activities has noticeably helped the interpersonality and social connections being formed for each individual who participated. The homeworks (eg.: Self Exhibition-collage, own worth emblem-collage etc.) and the active conduction of a diary has helped both to achieve results and have more involvement in the group.

**Image:**

**Figure:**
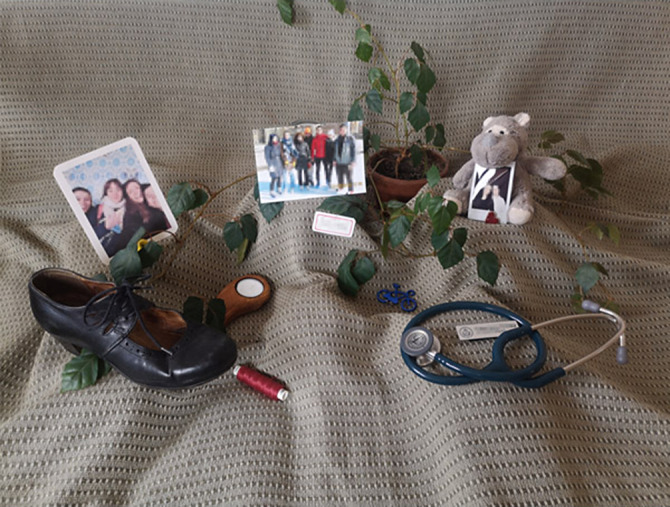

**Image 2:**

**Figure:**
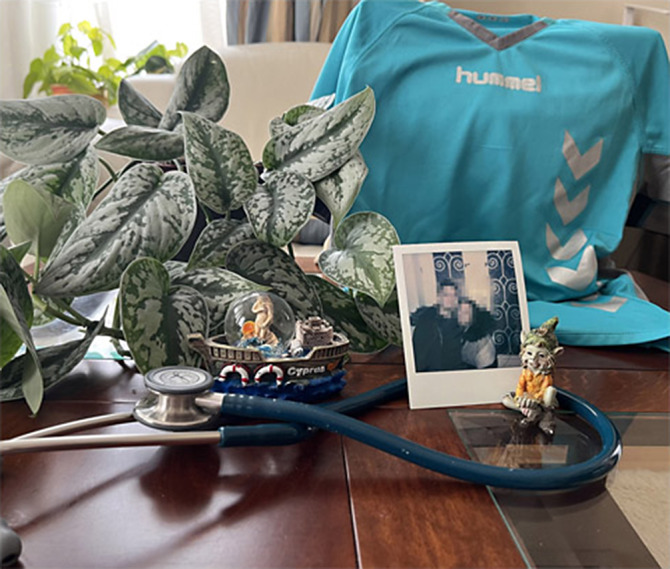

**Conclusions:**

Closing interviews are still in progress.

**Disclosure of Interest:**

None Declared

